# Rare diseases in ICD11: making rare diseases visible in health information systems through appropriate coding

**DOI:** 10.1186/s13023-015-0251-8

**Published:** 2015-03-26

**Authors:** Ségolène Aymé, Bertrand Bellet, Ana Rath

**Affiliations:** INSERM, US14 – Orphanet, Paris, France, Rare Diseases Platform, 96 rue Didot, Paris, France

**Keywords:** Rare diseases, Classification, Coding system

## Abstract

**Background:**

Because of their individual rarity, genetic diseases and other types of rare diseases are under-represented in healthcare coding systems; this contributes to a lack of ascertainment and recognition of their importance for healthcare planning and resource allocation, and prevents clinical research from being performed.

**Methods:**

Orphanet was given the task to develop an inventory of rare diseases and a classification system which could serve as a template to update International terminologies. When the World Health Organization (WHO) launched the revision process of the International Classification of Diseases (ICD), a Topic Advisory Group for rare diseases was established, managed by Orphanet and funded by the European Commission.

**Results:**

So far 5,400 rare diseases listed in the Orphanet database have an endorsed representation in the foundation layer of ICD-11, and are thus provided with a unique identifier in the Beta version of ICD-11, which is 10 times more than in ICD10. A rare disease linearization is also planned. The current beta version is open for public consultation and comments, and to be used for field testing. The adoption by the World Health Assembly is planned for 2017.

**Conclusions:**

The overall revision process was carried out with very limited means considering its scope, ambition and strategic significance, and experienced significant hurdles and setbacks. The lack of funding impacted the level of professionalism that could be attained. The contrast between the initially declared goals and the currently foreseen final product is disappointing. In the context of uncertainty around the outcome of the field testing and the potential willingness of countries to adopt this new version, the European Commission Expert Group on Rare Diseases adopted in November 2014 a recommendation for health care coding systems to consider using ORPHA codes in addition to ICD10 codes for rare diseases having no specific ICD10 codes. The Orphanet terminology, classifications and mappings with other terminologies are freely available at www.orphadata.org.

## Background

Because of their individual rarity, genetic diseases and other types of rare diseases are under-represented in healthcare coding systems; this contributes to a lack of ascertainment and recognition of their importance for healthcare planning and resource allocation, and prevents clinical research from being performed. This results in a poor understanding of their natural history and lack of knowledge of their epidemiology.

There are several thousands of rare diseases, disorders and conditions, the exact number being impossible to establish as it depends directly on the definition of not only what is the threshold for rarity but also on what is the definition of a clinical entity. The threshold for rarity is defined in some regions of the world in the context of regulations put in place to boost the development of therapies for rare diseases through incentives for Industry to invest [1]. It is based on the concept of prevalence or maximum number of patients in a region and ranges roughly from 1 in 10,000 to 1 in 1,000 persons. The definition in the European Union, as established by the Regulation (EC) N°141/2000 of the European Parliament and of the Council of 16 December 1999 on orphan medicinal products [2] is a prevalence of no more than 1 in 2′000.

Until recently there was no systematic effort to establish an inventory of rare disorders, except in the field of genetic defects where the Online Mendelian Inheritance in Man (OMIM) had started to document knowledge on genetic phenotypes as a proxy for genes, then on human genes when identified, as early as 1966 [3]. The compilation of an inventory of rare diseases, beyond genetic diseases, started in a systematic way in 1996, in the context of the rare disease database and knowledge base, Orphanet, established jointly by the French National Institute of Health and Medical Research (INSERM) and the French Ministry of Health [4] before becoming a Joint Action between the member countries of the European Union [5]. Orphanet not only collected information on rare diseases published in the scientific literature, but also classified them, from 2007 onwards, with a poly-hierarchy approach, each clinical entity being assigned an Orpha number. This effort was supported by the European Commission which not only co-financed Orphanet from 2001 onwards, but also established, in January 2004, a Rare Diseases Task Force with the mandate to contribute improve the codification of rare diseases, amongst other public health objectives.

This was instrumental in the decision to propose in 2007 that WHO make use of the Orphanet data to update ICD10 and design ICD11. This led to the establishment, in 2009, of a WHO Topic Advisory Group for Rare Diseases whose efforts and achievements are presented in this article.

### State of play of rare diseases coding in ICD10

An assessment of the number of rare clinical entities having a specific code in ICD10 can be derived from the effort carried out by Orphanet to cross-reference Orpha codes with ICD10 codes, starting from the Orphanet list of rare clinical entities defined as a clinically unique, distinct entity, whatever the number and nature of the causes, and following the European definition for rarity, i.e. a prevalence equal of no more than 1 in 2,000 in the general population of Europe. The cross-referencing is based on the 2010 online version of the ICD-10, but takes into account the official WHO updates endorsed in 2011 and 2012. In January 2015, among the over 6,954 clinical entities listed by Orphanet, 355 of them only have a unique specific code in ICD 10 and 162 can be specifically mapped to a set of ICD10 codes.

In fact the situation is complex as one ICD code sometimes corresponds to one Orpha code, but also one ICD code can correspond to a group of rare entities or to a group of both rare and non-rare entities. To increase rare disease representation in ICD-11, the objective is to expand the number of specific codes.

Orpha codes have also been mapped with UMLS terminologies [6]. In 2013 (UMLS-AB 2013 version) 32%, 23% and 14% of the Orphanet rare clinical entities had equivalent counterparts in SNOMED CT, MeSH, and MedDRA, respectively. These results demonstrate the necessity to expand the current coding systems so as to fairly represent entities which have a very significant impact on the healthcare systems and that are targets for innovation.

### The ICD revision process up-to-now

**Figure 1:**
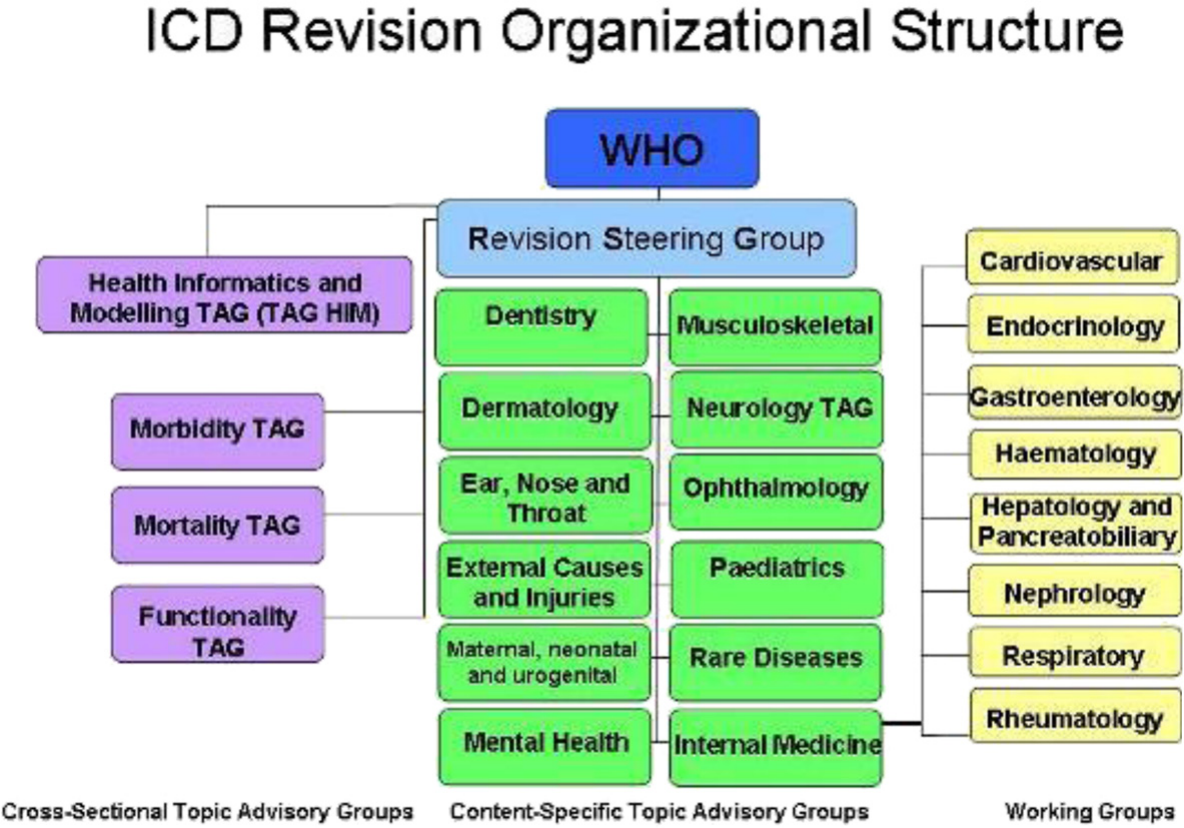
**ICD Revision organizational structure.**

The World Health Organization established various Topic Advisory Groups (TAGs) to serve as planning and coordinating advisory bodies in the update and revision process for specific areas (Figure 1). They were given the task of inviting working groups or individual experts to review their proposals. Each TAG is run by a chair and a managing editor [7]. A Revision Steering Group (RSG) including TAG chairs, representatives of the WHO-FIC (World Health Organization Family of International Classifications) Network, other invited terminologies, classification and public health experts, and relevant WHO officers were charged with overseeing the overall revision process through monthly teleconferences [8].

The revision process started in 2007. The RSG met for the first time in April 2007 to set out plans and define goals for the upcoming ICD-11. During the next meetings, the Health Informatics and Modelling (HIM) TAG was in charge of drafting the conceptual framework of entities and of developing Internet-based collaborative editing tools. A first project based on a wiki (*Lexwiki*) was rejected and replaced by a Protégé-based web-platform developed by a Stanford University team: the *ICD Collaborative Authoring Tool* (*iCAT*).

The official launch of the revision took place in September 2009 during a general meeting of TAG chairs and managing editors at the WHO headquarters in Geneva (‘i-Camp”). The revision proceeded in two phases. From September 2009 to May 2012, the TAGs worked to produce a first version of ICD-11 called the alpha-draft, using the iCAT. A public browser was opened in May 2011 to allow the public to view and then (from July 2011 on) to comment the alpha draft. From May 2012 onwards, the revision transitioned towards developing the beta-draft, which intends to be a relatively stable, reviewed draft fit for field testing use. The beta-draft was frozen in August 2014 to enable field trials to begin. Further comments are still collected on the public browser, and dispatched by the WHO to the relevant TAGs; corrections are from then now on implemented as a whole in periodic updates of the beta-draft. The first of those updates was published on early October 2014.

### General principles for ICD revision

The current ICD-10 classification is mono-hierarchical: meaning that every entity can figure only at one point in the classification. The historical rationale for this choice was to avoid double counting, since the ICD is primarily used as a statistical tool. This is a problem however for numerous diseases that can be associated with more than one body system, as chapters are broadly organised along them. In such cases, one system must then be given priority, and “exclusion notes” are put in the other relevant chapters to redirect users to the correct code.

The levels of detail in ICD-10 are limited to four: chapter (e.g. IV Endocrine, nutritional and metabolic diseases), block of codes (e.g. *E70-E90 Metabolic disorders*), three-character codes (e.g. *E70 Disorders of aromatic amino-acid metabolism*) and four-character codes (e.g. *E70.0 Classical phenylketonuria*). Additional details can be added only in clinical modifications used at the national level.

In the ICD-11, the classification is poly-hierarchical, and every entity is assigned a unique identifier. Diseases can figure in all places relevant for them in the tree structure; for instance, the several endocrine diseases associated with developmental anomalies will figure both in the endocrine and developmental anomalies chapters. This comprehensive tree-structure is called the *foundation*. It will be fully accessible in the electronic version of ICD11.

However, it is still necessary to keep the possibility of using a mono-hierarchical system: for space reasons in paper versions; to bundle data at specific levels for analysis; and most of all for statistics, to avoid double counting. Therefore, the ICD-11 will also feature *linearizations*, i.e. simplified versions arranged from a subset of the foundation to allow for a mono-hierarchical view of ICD-11 in which all selected items are **mutually exclusive** and **jointly exhaustive** for statistical purposes. Several linearizations are planned to accommodate various use cases: a joint linearization for mortality and morbidity statistics; two primary care linearizations, for low and high resource settings respectively; and several specialty linearizations, notably for dermatology, ophthalmology and rare diseases.

ICD-11 will feature up to seven levels of detail within every chapter. However, only the first three will be used in international comparisons; the additional four levels of detail will remain available for specific uses. It is thus important to ensure that important entities are visible in the first three levels.

In principle, every ICD-11 entity should be defined by elements of a content model [9], including a title, classification properties, a set of synonyms and inclusion terms, textual definitions and various associated properties. These datasets were designed to allow an ontological approach to ICD-11 entities [10]. The full set of parameters is described in Table 1.

**Table 1:** **List of field of the content model of ICD11**

1.	ICD entity title
2.	Classification properties
3.	Textual definitions
4.	Terms
5.	Body system/structure description
6.	Temporal properties
7.	Severity of subtype properties
8.	Manifestation properties
9.	Causal properties
10.	Functioning properties
11.	Specific condition properties
12.	Treatment properties
13.	Diagnostic criteria

Not all fields are mandatory: the only required parameters that those relevant to the description of the entity. The main focus has been set on filling the first seven parameters.

World Health Organization. *WHO ICD Revision Information Note n°4: ICD-11 Content Model*. Online on the WHO website: http://www.who.int/classifications/icd/revision/Information_Note_4_Content_Model_v.1.4Approved.pdf?ua=1.

## Methods

### Involvement of the rare diseases TAG

The Rare Diseases TAG was established in April 2007 to ensure that rare diseases would now be traceable in mortality and morbidity information systems (Table 2) .

**Table 2:** **WHO Rare Diseases Topic Advisory Group membership**

**Chair: Pr. Ségolène Aymé**	
**Managing editors: Dr. Ana Rath and Bertrand Bellet**	
**Representatives for specific areas:**	
**•**	Dr. Eduardo Castilla (Brazil)
**•**	Pr. Evgeny Ginter (Russia)
**•**	Dr. Stephen Groft (United States)
**•**	Pr. Julie McGaughran (Australia)
**•**	Ms. Kerry Innes (Australia)
**•**	Dr. Judith Allanson, afterwards replaced by Ms. Marion Williams (Canada)

The production of basic information on which to build the classification of rare diseases in ICD-11 was assigned to Orphanet. It contributed to the whole ICD revision process, considering that rare diseases involve all areas of medicine. Orphanet collects series of rare diseases classifications mainly based on scientific grounds (etiology and mechanism). To complement these classifications, a clinical in-house classification is developed to meet the needs of the clinicians. All the classifications, regularly updated as scientific knowledge evolves, can be viewed on the Orphanet website and have served as a basis to build the ICD-11 proposals by the Rare Diseases TAG.

For chapters where rare diseases feature prominently, or were dealt with early in the revision process, the Rare Diseases TAG proposed a whole revision of the structure together with the addition of rare diseases. These are the chapters for the blood and immune systems (ICD-10 chapter III), endocrine system, nutritional and metabolism (ch. IV), nervous system (ch. VI), respiratory system (ch. X), and developmental anomalies (ch. XVII).

For the other chapters, the new structure revision has been set up by the specific TAG for the body system and rare diseases were added into it by the Rare Diseases TAG. These are the chapters for infectious and parasitic diseases (ch. I), neoplasms (ch. II), eye (ICD-10 ch. VII), ear (ICD-10 ch. VIII), circulatory system (ch. IX), digestive system (ch. XI), skin (ch. XII), musculoskeletal and connective tissue (ch. XIII), genitourinary system (ch. XIV), pregnancy, childbirth and the puerperium (ch. XV), perinatal conditions (ch. XVI).

No proposal was made in the chapter on mental and behavioral disorders (ch. V). The reason is that the mental and behavioral disorders chapter is set up to allow the coding of levels of disabilities independently from their cause. Therefore it was not possible to establish new levels of coding to include rare diseases impacting on mental status and behavior, but conditions with disorders of intellectual developmental as a relevant clinical feature were listed in a dedicated grouping in the chapter for developmental anomalies. The inclusion of a new chapter for multi-systemic diseases was considered for a time and advocated for by the Rare Diseases TAG. Nevertheless, the WHO decided against its creation and struck out the in-work draft chapter in the iCAT on December 2011. The diseases that it would have contained were later redistributed into chapters for the individual body systems.

The WHO introduced during the writing of the alpha-draft a division of labor between TAGs when their areas of interest overlapped. The Rare Disease TAGs frequently experienced it, since rare diseases are found in every area of medicine. In such instances, the WHO assigned the primary responsibility for a chapter or section structure to a “primary TAG”, while other interested TAGs remained free to add and edit information. The Rare Diseases TAG was the primary TAG only for the section on developmental anomalies (ch. XVII).

### Revision procedures followed by the rare diseases TAG

**Figure 2:**
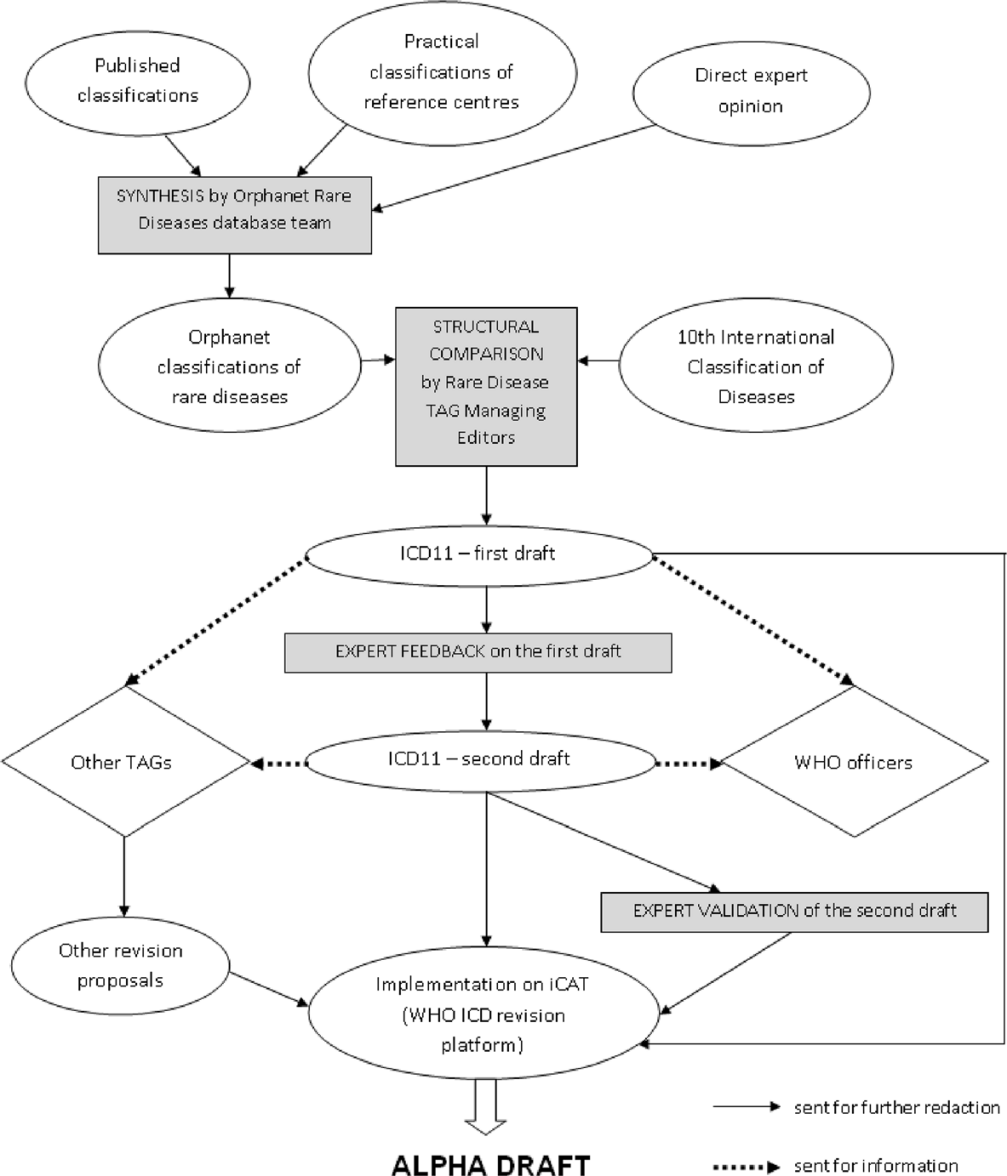
**Workflow followed by the Rare Diseases TAG for the preparation of the ICD11 Alpha-draft.**

For chapters where the Rare Diseases TAG proposed a full structural revision, the first stage was a systematic comparison between the extant ICD-10 and the Orphanet classifications of rare diseases. These classifications are produced and updated for the Orphanet database from a synthesis of medical and scientific publications, workshops with expert groups, and direct validation by specialists for restricted groups of disorders. The workflow is described in Figure 2.

General lines were defined. The chapters were organized primarily on a clinical basis, in line with the mortality and morbidity linearization. Etiology was considered afterwards for further detail. Genetic diseases were classified according to their clinical manifestations. However, they may be identified as genetic in the content model (from the Causal properties section), thus enabling to retrieve them separately. Rare diseases affecting several body systems were included in every relevant chapter, in accordance with the poly-hierarchical line of ICD11; but a main localization was suggested, to prepare for morbidity linearizations, according to the most severe involvement and/or the medical speciality most likely to be relied on for the management of the disease. In some cases, the choice is arbitrary because scientific evidence allows no sound decision. Tumors were systematically doubly classified in the chapter for neoplasms and in the chapter(s) dedicated to the involved body system(s). Morphologically-defined tumors, currently coded with ICD-O, were included in the draft. Constitutional and acquired disorders were regularly distinguished in the hierarchical structure of revised chapters. Categories inherited from the ICD-10 were systematically examined, and preserved as far as possible for the sake of historical continuity. However, the latter requirement was only clearly expressed at the beginning of the beta-draft phase; therefore, several drafts already corrected by experts had to be subsequently amended.

From this structural comparison, a draft for a new structure was written as a starting point to submit to working groups and experts. The drafts were sent by mail as PDF files, containing the rationale and general principles for the ICD revision and the revised structure, presented with synonyms and mappings with Orpha numbers. Working groups and experts for Europe were invited by the chair of the Rare Disease TAG. The other TAG members were responsible for disseminating the draft and invitation to contribute in their respective geographic areas: Brazil, Russia, United States, Canada, Australia, and to forward the collected feedback for analysis by the managing editor and assistant. From this feedback, a second, corrected draft was produced and sent for validation to the same experts.

For the revision of the chapter for developmental anomalies, for which the Rare Diseases TAG had primary responsibility, experts met in a conference held on December 2011 in Luxembourg to address the chapter’s structure after having been previously submitted a first draft and sent their preliminary feedback.

The implementation of the revised chapters in the iCAT has been variable across time. The first chapters were revised before the iCAT was functioning. Later, chapters were entered into the iCAT when the draft had been reviewed twice and validated by experts. Finally, chapters were entered into the ICAT directly at the stage of the first draft in order to allow other TAGs to comment them as well, and corrections submitted by experts made later as they were received.

Once the revision transitioned to the beta-phase, the Rare Disease TAG’s work shifted to ensuring that the rare diseases it had introduced into the ICD-11 structure were duly kept and accordingly classified, to finding accommodations with the requirements of other TAGs regarding their dedicated chapters, and to mapping iCAT and Orphanet identifiers to enable exchange of data between the two systems, notably in the prospect of populating of the ICD-11 content model with Orphanet data. Several hundreds of definitions for rare diseases have already been imported into the iCAT in that way. This mapping has to be regularly updated as the ICD-11 content is still evolving, notably by the reduction of duplicates and the redefinition of certain entities.

## Results

### Current situation of rare diseases in ICD 11

On 1 October 2014, 5,400 rare diseases listed in the Orphanet database have an endorsed representation in the foundation layer of the ICD-11, and are thus provided with a unique identifier in ICD-11, which is 10 times more than in ICD10. A mapping of those identifiers with ORPHA numbers has been established to allow data exchange and to ensure compatibility between the two information systems; it will need to be regularly updated as new frozen releases of the ICD-11 beta version are issued.

The content model is far from complete for most ICD-11 entities and in all likelihood will never be completed. The amount of data to be gathered is simply too great for the limited means available to the editors, both in terms of time and funding. Besides, keeping such a large repository of data up-to-date is bound to become quickly overwhelming, especially regarding genetic data which are rapidly evolving. The only realistic way to achieve the initial purpose of annotating ICD-11 entities with the planned set of properties at a professional level of quality would be for the WHO to establish long-time partnerships with stable institutions dedicated to gathering and managing the relevant biomedical data.

In the meantime, the focus on filling the whole of the content model has been scaled down: emphasis is now chiefly put on writing definitions for every disease in ICD-11. At least, this will allow the codes to be used without ambiguity. Around 4,000 rare diseases represented in ICAT have an associated definition so far: 2,600 were expressly created by the Rare Diseases TAG, the remaining 1,400 were imported or created by other groups and need yet to be reviewed by the Rare Diseases TAG. 1,400 definitions remain to be written.

The current state of the ICD-11 beta version is open to the public for consultation and comment on an online platform: http://apps.who.int/classifications/icd11/browse/f/en#/. Everybody is entitled to create an account with a profile and to post comments, which are filtered by the WHO and dispatched among the relevant TAGs. The TAGs advise on what is to be done and the corresponding corrections are then carried out by the WHO.

The beta version is now frozen, so that it may be stable enough to be used for practical tests in the field. Corrections are nonetheless still possible, but are implemented globally as packages: the beta version now evolves through successive releases rather than being in a state of continuous flux. The last frozen releases of the beta version occurred on 14 August 2014 and 1 October 2014.

The work of many TAGs, including the rare disease TAG, has been largely focused till now on elaborating the foundation layer. However, the importance of the linearizations is now clearly emerging. They will be the tools used in actual practice, each being specifically fitted to a specific use case, while retaining a mutual compatibility by their being derived from the common foundation layer. In some instance they can be regarded as a “patch”, allowing to work within a framework expressly tailored for a specific purpose, even when directly using the structure from the foundation layer would be inappropriate or too unwieldy. The most generally used linearization will doubtlessly be the Joint Mortality and Morbidity linearization, designed to establish international epidemiological statistics, according to the basic purpose of the ICD since its inception; but is not intended at all to be the only one. Our position is that linearizations should be developed for every specific use case requiring cross-comparison of institutional data from various origins. Specific interest groups should involve themselves in creating and publishing their linearizations, so that they may be clearly and reliably established, fixed and made known.

The Rare Disease TAG obtained the agreement of WHO for the creation of a linearization for rare diseases. It still remains to be practically developed. Other medical fields that are already planned to have their own specialty linearization are mental health, dermatology, musculoskeletal disease, neurology, paediatrics, occupational health and ophthalmology. The list is not limitative: other groups expressed their interest in having a specific tool, notably for allergology [11]. A linearization for medical genetics will certainly be required, the creation of which could be supported by the Rare Diseases TAG

## Discussion

The ICD 10th Edition was produced between 1982 and 1989 by annual revision conferences of a limited number of experts, and it was adopted in 1990 by the World Health Assembly. It was foreseen that 10-yearly (decennial) editions would continue as the method of revision with interim annual updates in between. When the 11th revision was due in 2000, a moratorium was suggested by the WHO Executive Board for the Secretariat to come up with a modern revision strategy in consultation with the Member States. The reason for this suggestion was the insufficient level of ICD-10 adoption by Member States: ICD was then used by only 96 Member States out of 191. The moratorium suggested better informatics support towards implementation. In the following years, the WHO addressed the implementation issues within the WHO Family of International Classifications (WHO FIC) Network and then formulated a revision strategy between 2003 and 2007. An International Revision Process Plan was developed to revise the classification content in line with advances in health sciences and to add the desired functionality using modern health informatics standards. The objective was to gather input from all stakeholders in an open and documented way. The revision process was initiated by a letter from the Director General of WHO to all Member States in April 2007.

The strategy adopted by WHO was extremely ambitious and difficult to implement. The advance of knowledge since the 1980s (when the basic structure of ICD-10 was elaborated) makes the need for a general overhaul quite obvious. It is simultaneously necessary to manage the transition from paper to electronic records and to make use of the wide array of new technical possibilities opened by the development of information technology over the last two decades. Hence the project of turning the old ICD-10, primarily thought and used as a reference tool for statistics, into an ICD-11, intended as a versatile reference tool for any setting requiring the use of an interoperable classification of diseases, with features reminiscent (and allowing the derivative development) of an encyclopedia and ontology. Unfortunately, the revision proceeded rather erratically, with very limited means considering its scope, ambition and strategic significance, and experienced significant hurdles and setbacks. The lack of funding impacted the level of professionalism that could be attained. The contrast between the initially declared goals and the currently foreseen final product is disappointing.

### Organizational issues

The collaboration between TAGs was left to personal initiatives only, as the TAGs were not given clear instructions on how they should actually work together. The Rare Disease TAG was especially well-placed to experience this issue, because its scope of intervention involved nearly every chapters of the ICD and naturally led it to interact with many of the other TAGs. In practice, the quality of those relationships was extremely variable: a few were efficient collaborations, some were more difficult but ultimately fruitful and resulted in agreement, but there were also points of utter divergence. The selected model of distributed terminology development [12] was more an experiment than a real organizational choice.

TAGs were constituted progressively during the revision process, without a formal process defining in advance their final number and exact scope of activities: the division of responsibilities between “primary” and “secondary” TAGs (see above) occurred only quite late during writing the alpha-draft. Since the TAGs started working at various periods, their level of understanding depended on the number of RSG meetings they could attend and their ability to explore the collaborative instruments. The TAGs that started working later on quite naturally ignored most of the basic rules agreed on orally but never explicitly written down in detail in a guidance document. Notably the full poly-hierarchy of ICD11, which allows all other TAGs create new hierarchies of diseases assigned to one specific ICD-10 chapter, was not understood by many TAGs. The absence of a restriction on levels of detail was not understood either. Several times, very rare but well-recorded diseases we had added into the new structure were discarded by the TAG for the relevant medical specialty as too rare to be of interest, or better represented as inclusion terms of bundled categories. This was not acceptable to us and caused many areas of friction. The necessity of unambiguous names once read out of context was not respected by most TAGs.

From our experience, the lack of agreement on and appropriation of the basic revision concepts and principles by the TAGs has been the source of many misunderstandings between TAGs. We opine that more attention should have been devoted to dealing collectively with such issues from the very start of the revision. Not enough time was spent on educating the medical experts involved in the TAGs on the requirements of an effective information system. Instead, the initial emphasis was rather put on educating TAG members on using collaborative tools. While there is of course no question that this was necessary, we argue that this was premature before the lineaments of this collaboration had been effectively settled. The resulting disagreements had to be solved later by time-consuming and sometimes difficult exchanges of mails, face-to-face meetings and teleconferences.

Many formal rules emerged during the revision process and were formalized only several years after the revision had begun. Consequently, the work carried out on many entities had to be repeated later to adapt the data to the finally decided format. In view of the limited means allotted to the revision, this produced an unnecessarily heavy workload as tasks had to be done and redone again and again, hence many delays, frustration and sometimes even anger with the whole process (resulting for instance in resignation). It is impossible here not to regret the waste of time that would have been saved by normalization from the start.

A few examples of this late formulation of rules are now given. Information notes formalizing various aspects, were produced only from the end of 2011 to 2013, so two years after the revision process had been launched, and four years after the preliminary discussions in 2007 [13]. Rules for the naming of diseases were not clear for many TAGs, despite the existence of a WHO style guide. In particular, many names were initially created that made sense only in the hierarchical framework the entity was included in, but which would have made little sense in an alphabetical list like the ICD-11 index. A template on how short definitions should be written has formalized by a dedicated WHO team in 2014 only [14].

On the whole, there appears in many TAGs to have been an understandable focus on medical issues but at the expense of those related to organizing knowledge into an information system. It shows that the background in information science of the revision organizers was unfortunately not transmitted efficiently enough to the experts participating into the TAGs. Much of their time probably went into appropriating the tools put at their disposal, when they actually did, since we are aware that a large amount of the proposals were finally implemented by WHO officials. Face-to-face meetings between each TAG and WHO officials proved to be absolutely necessary before the beta version could be reasonably put on line.

Alternatively, we think that fully acknowledging this division of the tasks between medical experts and information scientists would have been more efficient. Time and money could have been saved by gathering information from medical literature and taking advantage of the clinical modifications of ICD-10 used in various countries. Some elements of them may have been used during the revision, but no general analysis was ever presented. The TAGs missed here an opportunity to obtain a view of the existing needs of practicing coders on the field, to evaluate their consistency across countries and compare this feedback with the revision directions required by the advance of knowledge. From those data, a preliminary draft could have been produced by information professionals according to the general principles of ICD-11, as planned by the RSG. Then this draft would have been submitted to experts for discussion, and conferences held to establish a consensus. Finally information professionals would then have implemented the decided structure in the iCAT and submitted the result to the experts for validation.

The Rare Diseases TAG used this organization quite successfully. We also experienced several times the much greater added value of direct face-to-face discussion compared with mail or even phone calls, and wish to especially thank all experts that devoted some of their time to us in order to directly discuss the ICD-11 structure together.

### Conceptual issues

The revision process was supposed to produce a coherent classification, based on the current state of science, and adapted to the users’ needs. Unfortunately, the discussion on the structure happened only at the level of the Health Informatics and Modeling (HIM) TAG, at the conceptual level, and never between all the Revision Steering Group (RSG) members. None of the RSG meetings had a dedicated session for debating the basic principles guiding the revision of the structure. The result is that the current ICD-11 structure is at least as inconsistent as in ICD-10.

From the start of the revision, especially during the preliminary discussions from 2007 to 2009, the HIM TAG and RSG appear to have been much impressed by the social networking model on a large scale information project provided by Wikipedia, the free online encyclopedia that anyone can edit. It is definitely a successful model of collaborative work, with more than 4 600 000 articles in the English version to date, though at every possible level of advancement and quality. On the other hand, producing a generic all-public encyclopedia and a consistent reference tool for classification are quite different endeavors: while the former can thrive in a decentralized setting, as topics are highly diverse and consistency not necessarily an issue (with the possibility of cross references by internal links), the latter requires a much more thoroughly formalized framework. The bottom-to-top approach upheld by the RSG, especially in the early phases of the revision, might have been effective to produce a worldwide encyclopedia of diseases; but to produce a consistent classification, build a reference consensus and enable continuity with previous versions of ICD, a complementary top-to-bottom management was inescapable from the start. Nonetheless, the RSG left the TAGs very free (too free) to organize themselves and define the main lines of the structures they were building. An illustration of the result of this lack of direction is the notably different approaches adopted by the various TAGs. While such differing approaches may be commendable when corresponding medical specialties are considered in isolation, the global result appears to lack clearly defined classificatory principles. This makes it more difficult than needed to understand and may be a hurdle in the future for appropriation by coders. For instance, the chapter on diseases of the digestive system is mostly divided along anatomical lines (Table 3), when the chapter for neurology follows a more etiological approach [15] (Table 4). In the chapter for hematology, it was not possible to reach a consensus, so two different views coexist: one by classically described clinical groups (anemias, polycythemias, hemorrhagic diseases, thrombotic diseases etc.), corresponding to the Rare Diseases TAG draft, one based on etiology (iron deficiency, vitamin B12 deficiency, other nutritional and metabolic anemias, hemolytic anemias, etc.), following the requirements of the Hematology Working Group of the Internal Medicine TAG. Most of the entities are shared, but the structures are quite different, which will have consequences later for analysis depending on which one is chosen for aggregating data. According to the ICD revision procedures, a common classification should have been elaborated in the foundation layer of ICD-11; specific views are supposed to be represented at the linearization level, adapted for different use cases. As a final example, the chapter for circulatory diseases uses several approaches simultaneously, which makes it quite difficult to approach as a tree-structure (Table 5).

**Table 3:** **Structure of the classification of diseases of the digestive system in the Beta version of ICD11**

**▾**	**Diseases of the digestive system**
►	Digestive system disorders of fetus and newborn
►	Symtoms, signs and clinical findings involving the digestive system and abdomen
►	Disease and disorders of orofacial complex
►	Diseases of oesophagus
►	Disease of stomach
►	Disease of duodenum
►	Diverticular disease
►	Diseases of small intestine
►	Diseases of appendix
►	Diseases of large intestine
►	Diseases of anal canal
►	Diseases of liver
►	Diseases of gallbladder and biliary tract
►	Diseases of pancreas
►	Diseases of peritoneum
►	Hernia
►	Functional gastrointestinal disorders
►	Other diseases of the digestive system
►	Symptomsm, findings and clinical forms of the digestive system

**Table 4:** **Structure of the classification of diseases of the nervous system in the Beta version of ICD11**

**▾**	**Diseases of the nervous system**
►	Infections of the nervous system
►	Movement disorders
►	Neurological disorders with neurocognitive impairment as a major feature
►	Multiple sclerosis and other white matter disorders
►	Epilepsy and seizures
►	Headache disorders
►	Cerebrovascular diseases
►	Spinal cord disorders excluding trauma
►	Motor neuron diseases and related disorders
►	Disorders of nerve root, plexus and peripheral nerves
►	Diseases of neuromuscular junction and muscle
►	Cerebral palsy
►	Structural development anomalies of the nervous system
►	Syndromes with central anomalies of the nervous system
►	Disorders of cerebrospinal fluid pressure and flow
►	Injuries of the nervous system
►	Paraneoplastic and autoimmune disorders of the nervous system
►	Disorders of autonomic nervous system
►	Prion diseases
►	Disorders of consciousness
►	Other disorders of the nervous system
►	Functional clinical forms of the nervous system
►	Symptoms, sign and clinical findings involving the nervous system
►	Infections due to prions
►	Symptoms, findings and clinical forms of the nervous system

**Table 5:** **Structure of the classification of diseases of the circulatory system in the Beta version of ICD11**

▾	**Diseases of the circulatory system**
►	Neoplasms of the circulatory system
►	Cerebrovascular diseases
►	Symptoms and signs involving the circulatory system
►	Hypertensive diseases
►	Ischaemic heart diseases
►	Diseases of coronary artery
►	Pulmonary heart disease and diseases of pulmonary circulation
►	Pericarditis
►	Acute and subacute endocarditis
►	Heart valve disease
►	Rheumatic heart disease
►	Diseases of the myocardium
►	Cardiac arrhythmia
►	Congenital anomaly of heart and great vessels and related acquired abnormalities
►	Structural developmental anomalies of the pericardium
►	Structural development anomalies of the peripheral vascular system
►	Heart failure
►	Certain specified forms of heart disease
►	Complications and ill-defined descriptions of heart disease
►	Diseases of arteries and arterioles
►	Diseases of veins
►	Functional vascular disorders
►	Disorders of lymphatic vessels and lymph nodes
►	Certain specified disorders of the circulatory system
►	Hypotensions
►	Postprocedural disorders of circulatory system
►	Infections of the circulatory system
►	Symptoms, findings and clinical forms of the circulatory system

Multi-classified entities are often heterogeneously treated because of lack of rules on how to manage multi-classification of entities vs. classifying a disease and its particular manifestations. It was stated early that the dagger-and-asterisk system used in ICD-10 to represent primary and secondary involvement would be discontinued. Unfortunately, for a long time it was not made clear how it was to be replaced. Therefore, TAGs faced with the need to represent secondary involvements, or specific manifestations of a particular diseases, tried a variety of creative solutions resulting in great heterogeneity across chapters. The typical case was an infectious or multi-systemic disease created several times for every of its various manifestations, without them being associated anywhere under a common heading representing the disease as a whole, or the heading might have been created without including or ranging over subtypes for the various manifestations. This problem was acute for a long time but is now solved (largely by WHO officials) by establishing the correct parentings. Nevertheless, there are still lingering problems; for instance, in the digestive chapters, ulcers caused by Crohn disease or sarcoidosis have no formal link with Crohn disease or sarcoidosis.

### Technical issues

The iCAT tool had some limitations, although secondary compared to the organizational and conceptual issues described above. It must be made clear that those limitations are due to the lack of proper funding to sustain this ambitious process, after the main application initially developed had been turned down. The iCAT tool has been developed afterwards on a voluntary basis by a very small, but fortunately very skilled, reactive and committed team.

While effective and user-friendly for documenting diseases and managing hierarchies, the iCAT has not been designed to accommodate differences of views among editors in a multi-authored process. The choice was made to allow any editor to edit any entity; as a result, before editing rights were gradually restricted during the beta-draft phase, the final stage reflected the decision of the last editor, and could always be challenged again, resulting in great instability. The only way to point out differences was to leave editorial notes, as the tool allows every data to be annotated in free text. While this feature is clearly helpful for documenting the reason for changes and citing source, it proved unusable in practice to hold sustained discussions between editors, which have been therefore conducted mostly by private e-mail, and are therefore not available on the platform for consultation and citation.

The problem has been addressed in the ICD-11 beta browser, on which the general public is invited to consult and comment the forthcoming ICD-11: here a more centralized system has been set out, where comments are filtered by the WHO and dispatched to the relevant TAGs.

The inability of the iCAT to manage editorial rights and duties, coupled with the absence of clear rules, ended up creating a stressful situation where thousands of hours of work could be erased by another group modifying the iCAT content without any dialogue, without possibility to restore a previous version. This was counterproductive and disrespectful. It was a waste of resources ending up with a chaotic final product.

At the beginning of the revision, the platform’s internal search engine was quite inefficient and worked only on exact matches between character strings in entity titles. It did not effectively allow for a user to check whether an entity intended for creation by a TAG had not already been added before by another one. Since there are naturally many overlaps between the fields of medicine attributed to the various TAGs, this resulted in the production of a massive amount of duplicate entries, that had later to be detected (partly with the help of automatic scripts) and solved one by one by hand. This pruning was near to reaching completion in September 2014, as far as detected duplicates are concerned. It must be emphasized however that this was a frustrating and time-consuming task, which would have been at least partly avoided by delaying work on the iCAT before the search engine had been fixed to provide a reasonably effective safeguard against the creation of so many duplicates. There also remains the possibility of duplicates at a conceptual, not lexical level, i.e. the coexistence of entities that have a very similar scope but are named so differently that it is impossible to detect the problem by purely formal means. There is no other way for now other than relying on the expertise of the TAGs to deal with this issue.

## Conclusions

Currently, only a small fraction of rare diseases have codes in international nomenclatures, especially in ICD10, making it impossible to trace patients with rare diseases in health information systems on a national and international level. Having codes for each and every rare disease would help obtain a better knowledge of healthcare pathways and of their impact on specialized health care services and on budgets. The existence of specific codes for rare diseases are key to evaluate the performance of the European reference networks of centres of expertise for rare diseases which are about to be implemented.

It would also provide data for clinical research which is critically needed in this field, and would allow genomics activity, which is growing fast in developed countries, to be coded. Next generation sequencing data requires next-generation phenotyping [16]. The provided classifications need to offer a wide range of granularity level in order to code rare disease patients in different care settings, from primary care to centres of expertise for rare diseases, from clinical diagnosis up to genetic diagnosis

The revision of the International Classification of Diseases, which is the main instrument at world level, to code health events and draw statistics for International comparisons, is in its final stage. The current beta version is open for public consultation and comments, and for field testing use. The adoption by the World Health Assembly is planned for 2017. In the context of uncertainty around the outcome of the field testing and the potential willingness of countries to adopt this new version, the European Commission Expert Group on Rare Diseases adopted in November 2014 a recommendation for national health care coding systems to consider using ORPHA codes in addition to ICD10 codes when a rare disease has no specific ICD10 code [17]. As Orpha codes will be linked with ICD11 the switch from ICD10 to ICD11 will be made easier if decided.

It is not fair to the large community of patients with rare diseases, not to make their case visible in health information systems, and a solution should be adopted at least until the ICD11 is fully implemented in countries. An intermediate solution for the codification of rare diseases could be to add the ORPHA code to the current coding systems. The Orphanet terminology and classifications, and their mapping with other terminologies are freely available under Creative Commons Attribution-NoDerivs Licence, in computable user-friendly formats at www.orphadata.org (xml format or through the Orphanet Rare Diseases Ontology (ORDO) in OWL and boo formats). Two European countries, France and Germany, have already decided to adopt ORPHA codes as a complement to ICD10 and are in the pilot phase of implementation. Fourteen other European countries are considering the use of Orphacodes at national level and in some instances this is already a planned measure of their national plans/strategies for rare diseases [18]. ORPHA codes are already in use in some Italian regions covering half of the Italian population and the exploitation of the generated data demonstrate how useful this approach can be [19].

## Availability of supporting data

All classifications compiled by the Orphanet team are freely accessible at www.orphadata.org.

The beta version of ICD11 is accessible at http://apps.who.int/classifications/icd11/browse/f/en#/

Some committees and learned societies systematically received proposals to review:

- **For the EC Rare Diseases Task Force/ European Union Committee of Experts on Rare Diseases**: Mr. Andrew Devereau (United Kingdom), Dr. Shane McKee (UK), Dr. Domenica Taruscio (Italy), Mrs. Annet Sollie (Netherlands), Mr. Oscar Zurriaga (Spain)
- **For the European Medicines Agency’s Committee for Orphan Medicinal Products**: Dr. Jordi Llinares-Garcia
- **For the Paediatric Chairs of Canada** : Dr. Robert Armstrong, Dr. Jim Kellner, Dr. William Bingham, Dr. Cheryl Rockman-Greenberg, Dr. Guido Filler, Dr. Lennox Huang, Dr. Denis Daneman, Dr. Sarah Jones, Dr. Joseph Reisman, Dr. Bruno Piedboeuf, Dr. Harvey Guyda, Dr. Marc Girard, Dr. Herve Walti, Dr. Jonathan Kronick, Dr. Cathy Vardy, Dr. Aneal Khan, Dr. Bruno Maranda, Dr. Jonathan Kronick, Dr. Jane Gillis
- **For the National Center for Research Resources (United States)**: Dr. Barbara Alving
- **For the National Library of Medicine (United States):** Dr. Donald Lindbergh, Ms. Betsy Humphreys
- **For the National Institute of Health Clinical Center Hospital (United States)**: Dr. John Gallin

**Individual experts** were contacted according to their well-identified expertise or their role as coordinator of a research network. Those that returned comments are in bold:

- ***Developmental anomalies***: **Pr. Koenraad Devriendt** (Belgium), Pr. Albert E. Chudley (Canada), **Pr. Jane Evans** (Canada), Pr. Rachel Laframboise (Canada), **Dr. Marie Béland** (Canada), Dr. Nicolas Gilbert (Canada), **Pr. Alain Verloes** (France), **Dr. Martine Le Merrer** (France), **Dr. Agnès Bloch-Zupan** (France), **Pr. Damien Bonnet** (France), **Dr. Lucile Houyel** (France) **Pr. Raoul C. Hennekam** (Netherlands), Dr. Krystyna Chrzanowska (Poland), Pr. Malgorzata Krajewska-Walasek (Poland), Dr. Cristina Rusu (Romania), **Pr. Petr Novikov** (Russia), Dr. Adeeb Al-Omrani (Saudi Arabia), **Pr. Miguel del Campo Casanelles** (Spain), **Dr. Sixto García-Miñaúr** (Spain), **Pr. Albert Schinzel** (Switzerland), Dr. Aída Falcón (Venezuela), **Dr. Rodney Franklin** (United Kingdom), **Dr. Diana Wellesley** (United Kingdom), **Dr. Michele Lloyd-Puryear** (United States), **Dr. John Moeschler** (United States), **Pr. Robert H. Anderson** (United Kingdom), **Pr. Dian Donnai & Mr. Han Brunner** (Dyscerne), **Pr. Anthony Brookes** (Gen2Phen**)**, **Dr. Peter Robinson** (Human Phenotype Ontology), **Dr. Helen V. Firth** (Decipher), **Dr. Ester Garne** (Eurocat), **Pr. Ada Hamosh** (Online Mendelian Inheritance in Man)**, Dr. Nicole De Leeuw** (European Cytogeneticists Association Register of Unbalanced Chromosome Aberrations).
- ***Endocrinology***: Pr. Sabina Zaharieva (Bulgaria), Pr. Nikolai Botushanov (Bulgaria), **Dr. Celia Rodd** (Canada), Dr. David Stephure (Canada), Dr. Marta Šnajderová (Czech Republic), Dr. Vallo Tillmann (Estonia), Dr. Kirsti Näntö-Salonen (Finland), Pr. Philippe Chanson (France), Pr. Maïthé Tauber (France), Dr. Felix Beuschlein (Germany), Dr. Catherine Dacou-Voutetakis (Greece), **Dr. Evangeline Vasilatou** (Greece), Dr. Marco Cappa (Italy), Pr. Vincenzo Trischitta (Italy), Pr. Sebastiano Filetti (Italy), Pr. Jolanta Sykut-Cegielska (Poland), Dr. Eusebiu Zbranca (Romania), **Dr. Anatoly Tiulpakov** (Russia), **Pr. Susana Webb Youdale** (Spain), Pr. Angel Carrascosa (Spain), Dr. Rüveyde Bundak (Turkey), Pr. Julian Davis (United Kingdom), Pr. Gary Butler (United Kingdom).
- ***Gastroenterology, Nephrology, Diabetes****:* Dr. Griffin Rogers (National Institute of Diabetes, Digestive and Kidney Diseases, United States).
- ***Haematology***: Dr. Dieter Lutz (Austria), Dr. Henrik Birgens (Denmark), Dr. Vessela Goranova (Bulgaria), Pr. Frédéric Galacteros (France), Pr. Hubert Schrezenmeier (Germany), Dr. Panayiotis Tsaftaridis (Greece), **Dr. Paquita Nurden** (France), Pr. Robin Foà (Italy), Dr. Ecaterina Hanganu (Romania), **Dr. Michel Duchosal** (Switzerland), Pr. Teoman Soysal (Turkey), **M.D. Inderjeet Dokal** (United Kingdom), Dr. Michael Leaker (Canada), **Pr. Andrew Spencer** (Australia) **Pr. Joan Lluís Vives Corrons** (ENERCA), Pr. Flora Peyvandi (Rare Blood Diseases Database), **Dr. Donna DiMichele** (National Heart, Lung and Blood Institute, United States).
- ***Immunology***: Pr. Mariana Murdjeva (Bulgaria), **Dr. Reza Alizadehfar** (Canada), Dr. Christine McCusker (Canada), Dr. Nada Jabado (Canada), Dr. Raivo Uibo (Estonia), Pr. Alain Fischer (France), Pr. Hans-Hartmut Peter (Germany), Pr. C. G. M. Kallenberg (Netherlands), Pr. **Ewa Bernatowska** (Poland), Dr. Carlos Vasconcelos (Portugal), **Dr. Eugen Carasevici** (Romania), **Dr. Rahim Chaitov** (Russia), Dr. Miguel Lopez Botet (Spain), Dr. Cecilia Muñoz Calleja (Spain), Dr. Nuria Matamoros Florí (Spain), **Pr. Günnur Deniz** (Turkey), **Pr. Luigi Daniele Notarangelo** (United States), **Dr. Mort Cowan** (United States), Dr. Linda M. Griffith (United States), **Dr. Donna DiMichele** (United States), **Dr. Henry Chang** (United States), **Pr. Mauno Vihinen** (Information Network for Immunodeficiencies), **Pr. Stephan H. E. Kaufmann** (European Federation of Immunological Societies)**,** Pr. Amos Etzioni (European Society for Immunodeficiencies), **David Watters & Johan** Prévot (International Patient Organisation for Primary Immunodeficiencies)**, Pr. Helen Chapel & Pr. Mimi Tang** (International Union of Immunological Societies).
- ***Inborn Errors of Metabolism***: Dr. Wolfgang Sperl (Austria), Pr. George van den Berghe (Belgium), Pr. François Eyskens (Belgium), **Dr. Jiří Zeman** (Czech Republic), **Pr. Pascale de Lonlay** (France), **Pr. Jean-Marie Saudubray** (France), Pr. Udo Wendel (Germany), **Dr. Carlo Dionisi-Vici** (Italy), **Pr. Jan Smeitink** (Netherlands), Pr. Paula Grigorescu-Sido (Romania), **Dr. Ekaterina Zakharova** (Russia), **Dr. Teresa Pàmpols Ros** (Spain), Dr. Ali Dursun (Turkey), Dr. Eduardo Wraith (United Kingdom), Pr. James V. Leonard (United Kingdom), **Dr. Sara Copeland** (United States), **Dr. Susan Winter** (United States), **Dr. Neil Buist** (United States), : Dr. Mick Henderson & Dr. James Bonham (ERNDIM), Dr. Bryan Winchester (ESGLD), Mr. H. Stroomer (Eumitocombat), **Pr. Gert Matthijs** (Euroglycanet), Pr. Manuel Palacín Prieto (EUGINDAT), **Pr. Jean-Charles Deybach & Dr. Aasne Karine Aarsand** (European Porphyria Network), Pr. Paula Saftig (HUE-MAN), Mrs. Rosy Engelen (Mitocircle), **Pr. Guy Besley** (Society for the study of inborn errors of metabolism), Dr. John Walter (British Inherited Metabolic Diseases Group), **Dr. Robert Naviaux** (United Mitochondrial Diseases Foundation).
- ***Neurology***: Pr. Franz Aichner (Austria), Dr. Pavel Balabanov (Bulgaria), Pr. Veneta Bojinova (Bulgaria), Pr. Paraskeva Stamenova (Bulgaria), **Dr. Michael Shevell** (Canada), Dr. Myriam Srour (Canada), Dr. Karen Barlow (Canada), Pr. Tat’ána Maříková (Czech Republic), Dr. John Vissing (Denmark), Dr. Anneli Kolk (Estonia), Dr. Valentin Sander (Estonia), Dr. Sulev Haldre (Estonia), Dr. Katrin Gross-Paju (Estonia), Dr. Tiina Talvik (Estonia), Dr. Aki Hietaharju (Finland), Pr. Juha E. Jääskeläinen (Finland), **Dr. Tiina Tyni** (Finland), Pr. Philippe Coubes (France), Pr. Olivier Dulac (France), Pr. Bruno Eymard (France), **Pr. Bertrand Fontaine** (France), Dr. Thomas Klockgether (Germany), Pr. Thomas Voit (Germany), **Dr. Sotiria Mastroyianni** (Greece), Dr. Sámual Komoly (Hungary), Dr. Richard Petty (Ireland & United Kingdom), Dr. Enrico Bertini (Italy), Dr. Luciano Merlini (Italy), Pr. Francesco Muntoni (Italy), **Dr. Enza Maria Valente** (Italy), Pr. Valmantas Budrys (Lithuania), Pr. Milda Endziniene (Lithuania), Pr. Marianne De Wisser (Netherlands), Pr. Felicia Stefanache (Romania), Pr**. Sergei Illarioshkin** (Russia), Pr. Jaime Campos-Castello (Spain), Dr. Hand Jung (Switzerland), Pr. Piraye Serdaroglu (Turkey), Pr. Volker Straub & Pr. Kate Bushby (TREAT-NMD), **Dr. Solomon Moshe** (International League Against Epilepsy), **Pr. Barbara Wilson** (Encephalitis Society), **Pr. José Ferro** (European Neurological Society), Dr. Robin Grant (European Association of Neurooncology), Pr. Robert Ouvrier (International Child Neurology Association), **Dr. Alexandra Dürr** (Spatax)**, Pr. Alastair Compston** (Association of British Neurologists), Dr. Petra Kauffman (National Institute of Neurological Disorders and Stroke, United States).
- ***Nutritional diseases***: Dr. Jeff Critch (Canada), Dr. Stephanie Atkinson (Canada), Dr. Veronique Anne Pelletier (Canada), Pr. Victor Tutelian (Russia).
- ***Respiratory diseases****:* Dr. Panayiotis Yiallouros (Cyprus), Dr. Tacjana Pressler (Denmark), Dr. Maire Vasar (Estonia), **Dr. Rain Jõgi** (Estonia), **Dr. Katriina Kahlos** (Finland), **Pr. Jean-François Cordier** (France), Pr. Joachim Müller-Quernheim (Germany), Pr. Renato Cutrera (Italy), **Pr. Edvardas Danila** (Lithuania), Pr. C. K. van der Ent (Netherlands), Pr. João Carlos Winck (Portugal), Dr. Antonio Román Broto (Spain), Dr. Julio Ancochea Bermúdez (Spain), Dr. Juan José Morell Bernabé (Spain), **Dr. Eeva Piitulainen** (Sweden), **Dr. Romain Lazor** (Switzerland), **Dr. Jeffrey Kaufman** (United States), **Dr. Michael Schechter** (United States), **Dr. Edwin K. Silverman** (United States), **Dr. Ivy Dunbar** (United States), **Pr. Annick Clément** (Reference centre for rare respiratory diseases, Hôpital d’enfants Armand Trousseau), **Pr. Wim Wuyts** (Interstitial lung disease program - Leuven, Belgium), **Dr. Colin Wallis** (British Paediatric Respiratory Society), Dr. Stephen Conellan (British Thoracic Society)
- ***Infectiology and Allergology****:* Dr. Anthony Fauci (National institute of Allergy and Infectious Diseases, United States)
- ***Oncology***: **Dr. Gemma Gatta** (Rarecare).
- ***Paediatrics***: Dr. Alan Guttmacher (National Institute of Child Health and Human Development, United States)
- ***Rheumatology and Dermatology****:* Dr. Stephen Katz (National Institute of Arthritis, Musculoskeletal and Skin Diseases, United States)

### Special thanks

We want to warmly thank some WHO staff for their practical help with many project issues: Robert Jakob, Molly Meri Robinson Nicol, Can Celik. We always had appropriate and timely feed-back from the team which developed the iCAT: Tania Tudorache, Csongor Nyulas, Samson Tu, from Stanford University.

We had helpful discussions and collaborative work with some TAGs, especially:

- Robert Chalmers (Dermatology TAG)
- Linda Edwards (Paediatrics TAG)
- Julie Rust and Megan Cumerlato (Internal Medicine TAG)
- Rodney Franklin (Cardiology WG of the Internal Medicine TAG)

## Abbreviations

WHO: World Health Organisation
TAG: Topic Advisory Group
ICD: International Classification of Diseases
ICD-11: 11th version of ICD
iCAT: Collaborative authoring tool for ICD11
RSG: Revision Steering Group
UMLS: Unified medical language standards
MeSH: Medical subjects headings
MedDRA: Highly specific standardised medical terminology to facilitate sharing of regulatory information internationally for medical products used by humans
